# Overexpressing neurogenic differentiation factor 1 in Müller cells improves retinal function after optic nerve crush injury in adult mice

**DOI:** 10.4103/NRR.NRR-D-24-01144

**Published:** 2025-09-03

**Authors:** Liting Zhong, Dashuang Yang, Xiu Han, Haiyang Cheng, Di Xu, Wen Li, Gong Chen, Ying Xu

**Affiliations:** 1 Guangdong-Hongkong-Macau Institute of CNS Regeneration, Key Laboratory of CNS Regeneration (Ministry of Education), Guangdong Key Laboratory of Non-human Primate Research, Jinan University, Guangzhou, Guangdong Province, China; 2 Co-Innovation Center of Neuroregeneration, Nantong University, Nantong, Jiangsu Province, China

**Keywords:** glial cell-derived neurotrophic factor, inflammation, Müller cell, NeuroD1, optic nerve injury, retinal ganglion cell, synaptic connection

## Abstract

Optic nerve injury leads to axonal degeneration and the death of retinal ganglion cells, which ultimately causes vision loss. Notably, current treatments are limited. In the present study, we explored whether neurogenic differentiation factor 1 (NeuroD1 or ND1) overexpression in retinal Müller cells may repair the retina after optic nerve crush in mice. Adult mice were subjected to optic nerve crush followed by intravitreal AAV-7m8-GFAP-GFP-ND1 virus injection. Immunofluorescent staining, multi-electrode array recording, electroretinogram, and visual behavior tests were then performed to examine retinal and optic nerve structure and retinal function at various post-optic nerve crush and virus injection times. Western blot analysis and quantitative reverse transcription polymerase chain reaction were performed to explore the possible mechanisms. Compared with the control virus, specific overexpression of ND1 in Müller cells greatly improved the light responses of retinal ganglion cells and retinal neurons in optic nerve crush–injured mice as early as 1–2 weeks post-virus injection and lasted for up to 4 weeks. Neuronal survival in the ganglion cell layer and synaptic connections in the inner retina were slightly improved at 2 weeks; however, visual behavior, retinal ganglion cell survival, and optic nerve structure were not improved. ND1 transiently enhanced glial cell–derived neurotrophic factor expression in the optic nerve crush–injured retina but hardly inhibited retinal inflammation within 2 weeks. Together, our data indicate that ND1 overexpression in Müller cells improves retinal function in the optic nerve crush–injured retina, and suggest that its neuroprotective effect may be caused by enhanced glial cell–derived neurotrophic factor release.

## Graphical abstract

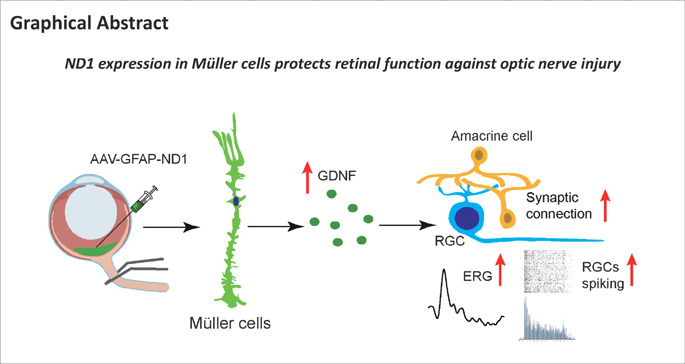

## Introduction

Damage to the optic nerve, which commonly results from traumatic optic neuropathy or optic nerve degeneration in glaucoma, triggers axonal degeneration and the gradual loss of retinal ganglion cells (RGCs). It can lead to permanent vision loss by disrupting the transmission of visual information to the brain. Clinical treatment for traumatic optic neuropathy typically involves high-dose corticosteroids and optic canal decompression (Yu-Wai-Man and Griffiths, 2013); however, outcomes are often unsatisfactory, and the balance between the clinical benefits and risks remains under debate (Steinsapir and Goldberg, 2011; Wladis et al., 2021). Despite ongoing research, the protection of degenerating axons and RGC soma in glaucoma remains unresolved (Zhao et al., 2023). Hence, there is an urgent need to explore novel therapeutic approaches for optic nerve injury.

Müller cells are crucial retinal glial cells that provide functional, structural, and metabolic support to maintain retinal homeostasis (Devoldere et al., 2019). Their neuroprotective and regenerative capabilities make them a crucial target in retinal therapy. Müller cells promote the survival of retinal neurons by releasing cytokines, growth factors, and neurotrophic factors (Devoldere et al., 2019). Furthermore, they exhibit stem-cell-like properties; they are capable of regenerating new retinas in lower vertebrates following injury (Goldman, 2014; Jui and Goldman, 2024) and can differentiate into multiple types of retinal neurons in adult rodents via various mechanisms (Gao et al., 2021). Targeting Müller cells thus represents a promising strategy for treating optic nerve injury.

Neurogenic differentiation factor 1 (NeuroD1 or ND1) is a key transcription factor that is involved in retinal development (Cho et al., 2007; Liu et al., 2008). It exhibits neuroprotective effects when overexpressed in astrocytes, and reprograms glia into neurons in the central nervous system (Chen et al., 2020; Liu et al., 2020). ND1 overexpression can also repair brain tissue in various rodent and primate disease models (Guo et al., 2014; Wu et al., 2020, 2025). In normal mouse retinas, we have observed that ND1 overexpression reprograms Müller cells into various types of retinal neurons, including ganglion cells, amacrine cells, horizontal cells, and photoreceptors (Xu et al., 2023). Furthermore, studies have demonstrated that ND1 overexpression inhibits inflammation in the injured mouse cortex (Zhang et al., 2020), indicating its neuroprotective potential. Thus, in the present study, we aimed to determine whether overexpressing ND1 in Müller cells protects retinal neurons following optic nerve injury, and investigated the underlying mechanisms involved.

## Methods

### Animals

Male wild-type (C57BL/6J) mice (specific pathogen-free grade; body weight, **~**22 g; age, 7 weeks) were purchased from Liaoning Changsheng Biotechnology Co., Ltd. (Benxi, China; License No. SCXK (Liao) 2020-0001). The mice were maintained under standard conditions, including a 12-hour dark/light cycle with free access to water and food. Experimental procedures adhered to the ARVO Statement for the Use of Animals in Ophthalmic and Visual Research, and received approval from the Qualified Ethics Committee of Jinan University on July 6, 2021 (approval No. IACUC-20210706-01), and were conducted in accordance with the National Institutes of Health Guide for the Care and Use of Laboratory Animals (8^th^ ed., National Research Council, 2011). Every effort was made to reduce the number of animals used and minimize their discomfort.

### Optic nerve crush surgery

For the optic nerve crush (ONC) procedure, mice were anesthetized with an intraperitoneal injection of 1.25% tribromoethanol (0.2 mL/10 g body weight, Merck KGaA, Darmstadt, Germany). Under a dissection microscope, the conjunctiva ring beneath the cornea–scleral junction was incised to expose the optic nerve. Subsequently, at approximately 0.5 mm behind the globe, the optic nerve was clamped with 100 g hemostatic forceps for 3 seconds. Successful crushing was generally indicated by pupil dilation. After surgery, the eyeball was repositioned, and antibiotic eye gel was applied to prevent infection. Because the optic nerve projects bilaterally to both brain regions, we performed bilateral surgery on the optic nerve to avoid the interference of input from the uninjured side. This procedure allowed us to accurately evaluate visually evoked potentials from the visual cortex, which is an important indicator of optic nerve repair. It also avoided large variations among animals, thereby reducing the number of animals used.

### Virus construct and intravitreal injection

Two viruses were constructed by OBiO Technology (Shanghai, China). They included AAV7m8-GFAP-EGFP-2A-MCS-3FLAG (green fluorescent protein [GFP] control virus, titer of 5.31 × 10^12^ vg/mL) and AAV7m8-GFAP::EGFP-2A-ND1 (ND1 virus, titer of 3.2 × 10^12^ vg/mL). The glial fibrillary acidic protein (GFAP) promoter was the 681-bp gfaABC1D, derived from the gfa2 promoter with a length of 2.2 kb (Lee et al., 2008).

For the virus injection, mice were anesthetized with 1.25% tribromoethanol (0.2 mL/10 g body weight) and their pupils were dilated using 0.5% tropicamide solution (Santen Pharmaceutical Co., Osaka, Japan). A sharp 30-gauge needle was used to puncture the cornea before 1.2 μL of virus was injected into the vitreous using a 34-gauge blunt-end needle. The needle was retained in the vitreous for an additional 10 s before its slow removal, followed by the application of antibiotic gel. For a direct comparison of the effects of the virus on retinal structure and function, the GFP virus was administered to one eye and the ND1 virus was administered to the other eye of experimental animals. For behavioral tests involving both eyes and for RNA/protein extraction that required substantial retinal tissue, the same virus was injected into both eyes, and the samples from animals injected with different viruses were compared.

To label the optic nerve fibers, cholera toxin β (CTB) subunit conjugated with Alexa 647 (C34778; Thermo Fisher Scientific, Waltham, MA, USA) (1 μL, 1 μg/μL) was intravitreally injected 3 days before the animal was sacrificed at 1 or 2 weeks post-virus injection (wpi); ONC was performed 1 day before the virus injection. Animals exhibiting inflammation or cataracts were excluded from subsequent experiments.

### Visual behavior tests

Mouse visual behavior was assessed using a black–white transition system (custom-made by Metronet Technology Ltd., Beijing, China) and an optomotor system, as previously described (Liu et al., 2021). The black–white transition system evaluates luminance discrimination ability. Briefly, each mouse was placed in an illuminated white box connected to a black box, with free movement allowed between them. The movement was recorded by infrared cameras mounted atop each box, and the time spent in the black box during a 5-minute test was analyzed using Noldus EthoVision XT 8.0 software (Doldus, Wageningen, Netherlands).

The optomotor test was used to assess visual acuity via the optomotor reflex to moving gratings with fine spatial frequency. Mice were placed on a high platform that was surrounded by four computer screens. The screens displayed vertical sine gratings that rotated either clockwise or counterclockwise; these were programmed using MATLAB software (MATLAB 8.0, MathWorks, Natick, MA, USA). The gratings had a 100% contrast, moved at 12 cycles/s, and increased in spatial frequency from 0.1 to 0.6 cycles/degree. The mice tracked the gratings by moving their heads (optomotor reflex) as long as they were visible. Head movements were recorded, and the maximum spatial frequency that elicited an optomotor reflex was manually recorded as the visual acuity.

### Multi-electrode array recordings and data analysis

We used multi-electrode array (MEA) recordings to assess the light-induced spiking of individual RGCs from whole-mount retinas, as previously detailed in Liu et al. (2021). In brief, a flattened retina was gently pressed onto an 8 × 8 MEA array (Alpha MED Scientific, Inc., Osaka, Japan), with the ganglion cells pressed down onto the electrodes. The array with the retina was then moved to a recording stage connected to an amplifier (MED64, (Alpha MED Scientific, Inc.). The tissue was continuously perfused with oxygenated Ames’ medium (Sigma-Aldrich, Shanghai, China) at a flow rate of approximately 4 mL/min; the temperature of the bath was maintained at 31–33°C. A full-field white flash was projected onto the retina using a white light-emitting diode (Thorlabs, Newton, NJ, USA) controlled by the MED64 amplifier. The light stimulus protocol consisted of 30 repeats of 10 seconds of stimuli, each of which consisted of 2 seconds light OFF, 2 seconds light ON, and 6 seconds light OFF. The flash intensity was set to 4.68 × 10^7^ (log 7.67) photons/μm^2^/s, which was sufficient to saturate the retina.

Data were recorded using MED64 Mobius software (Alpha MED Scientific, Inc.) and processed using the Offline Sorter (Plexon, Dallas, TX, USA) to isolate single-unit spike trains. The data were then further analyzed using Spike 2 (Version 8; CED, Cambridge, UK), R (version 3.5.1), and Matlab (MathWorks). For each spike-sorted unit, the peristimulus time histogram (PSTH) and raster plot with a width of 10 ms were generated and analyzed. Spontaneous spiking was quantified as the average response within 2 seconds before light onset (ON) or offset (OFF). Light responses were quantified as the average or peak spike rate during the first 2 seconds of ON, or OFF, or the average of both (for ON-OFF), minus the spontaneous spiking. To assess the speed of signal transmission, latency was measured as the time from light stimuli to the PSTH peak. To evaluate the transfer efficiency of visual information, the signal-to-noise ratio was calculated as the light response of each cell divided by the standard deviation of its spontaneous firing (Barrett et al., 2015; Liu et al., 2021).

### Electroretinogram recording

To evaluate the retinal light response, we conducted electroretinogram recordings on dark-adapted animals using the RETI-scan system (Roland Consult, Brandenburg, Germany), as previously described (Liu et al., 2021). Briefly, dark-adapted mice were anesthetized with tribromoethanol and their pupils were dilated using tropicamide (0.5%). After connecting the active and reference electrodes, the mice were exposed to full-field green flashes with intensities of 0.01, 0.1, and 3.0 cd·s/m^2^ to record the scotopic responses. Next, they were light-adapted with green light of 20 cd/m^2^ for 5 minutes, and the photopic responses were recorded at green flashes with intensities of 3.0 and 10.0 cd·s/m^2^. The amplitudes of the a-wave (the group response of photoreceptors), b-wave (bipolar cell response), photopic negative response (PhNR; ganglion cells), and oscillatory potentials (OPs; amacrine cells) were measured using RETI software after being low-pass filtered at 50 Hz. The OPs were analyzed after applying a band-pass filter of 70–450 Hz, and peak-to-peak amplitudes were measured for each OP peak. For each mouse, the average response of the two eyes was considered as a single data point.

### Tissue processing, immunohistochemistry, and image processing

To investigate the effects of virus infection, animals were euthanized by anesthesia overdose at various time points post-virus injection, and their eyes were enucleated and fixed in 4% paraformaldehyde at room temperature for 30 minutes. To process retinal slices, the eyecups with lenses were cleaned with phosphate-buffered saline (PBS) and cryo-protected overnight at 4°C in 0.01 M PBS containing 30% sucrose. They were then embedded in optimal cutting temperature compound (Tissue Tek, Torrance, CA, USA). Next, the retinas and optic nerves were cryo-sectioned using a microtome (Leica Microsystems, Buenos Aires, Argentina) at a thickness of 16 μm and 10 μm, respectively, longitudinally through the optic nerve head. The sections were then mounted on glass slides for further processing. To process whole-mount retina after paraformaldehyde fixation, the retina was separated from all other layers before being either stored in PBS at 4°C, or flattened on a glass slide and sealed with antifade mounting medium.

For immunostaining, retinal sections were incubated with blocking buffer (0.01 M PBS containing 0.3% Triton X-100, 10% normal donkey serum, and 3% bovine serum albumin) for 1 hour, followed by overnight incubation with primary antibodies at 4°C. The sections were then washed before being incubated with secondary antibodies for 2 hours at room temperature. Subsequently, they were washed again, mounted, and coverslipped. For nuclear staining, the sections were incubated with 4′,6-diamidino-2-phenylindole solution (Electron Microscopy Sciences, Hatfield, PA, USA) at room temperature for 5 minutes before mounting. The antibody information is listed in **Table 1**.

**Table 1:** Antibodies used for immunofluorescent staining

Antibody	Supplier	Cat#	RRID	Dilution
**Primary antibody**				
Chicken anti-GFP	Abcam, Cambridge, UK	ab13970	AB_300798	1:1000
Mouse anti-PSD95	Abcam	ab13552	AB_300453	1:500
Rabbit anti-Calretinin	Swant AG, St. Gallen, Switzerland	CR7697	AB_2619710	1:1000
Rabbit anti-Iba1	FUJIFILM WAKO SHIBAYAGI, Shibayagi, Japan	019-19741	AB_839504	1:1000
Rabbit anti-NEFM	Huabio, Hangzhou, Zhejiang, China	ET1703-57	AB_3070413	1:200
Rabbit anti-NeuN	Abcam	ab177487	AB_2532109	1:1000
Goat anti-ChAT	Millipore, Bedford, MA, USA	AB144P	AB_2079751	1:1000
Rabbit anti-ND1	Invitrogen	PA5-78075	AB_2736208	1:1000
Rabbit anti-RBPMS	Genetex, Irvine, CA, USA	GTX118619	AB_10720427	1:1000
Rabbit anti-Sox9	Millipore, Bedford, MA, USA	ab5535	AB_2239761	1:1000
Rat anti-GFAP	Invitrogen, Carsbad, CA, USA	13-0300	AB_86543	1:1000
**Secondary antibody**				
Donkey anti-mouse Alexa Fluor 546	Invitrogen	A10036	AB_11180613	1:1000
Donkey anti-rabbit Alexa Fluor 594	Jackson ImmunoResearch, West Grove, PA, USA	711-585-152	AB_2340621	1:1000
Donkey anti-rabbit Alexa Fluor 647	Invitrogen	A-31573	AB_2536183	1:1000
Goat anti-chicken Alexa Fluor 488	Jackson ImmunoResearch	103-545-155	AB_2337390	1:1000
Goat anti-rat Alexa Fluor 594	Invitrogen	A-21209	AB_2535795	1:1000

ChAT: Choline acetyltransferase; GFAP: glial fibrillary acidic protein; GFP: green fluorescent protein; Iba1: ionized calcium binding adaptor molecule 1; ND1: neurogenic differentiation factor 1; NEFM: neurofilament medium polypeptide; NeuN: neuronal nuclei; PSD95: post-synaptic density-95; RBPMS: RNA-binding protein with multiple splicing.

Fluorescent images were captured using either a fluorescence microscope or a confocal microscope (Carl Zeiss, Oberkochen, Germany). Images of the retinal sections were taken 320 μm away from the center of the optic nerve (or where GFP signals were detected). To measure the thickness of retinal layers, vertical lines were drawn across retinal sections at the left, middle, and right sites of each image. ImageJ 1.53t software (National Institutes of Health, Bethesda, MD, USA) was used to measure the lengths of these lines, which were then averaged to obtain a single value per image. To estimate the survival of RGCs, RNA-binding protein with multiple splicing-positive (RBPMS^+^; 1:1000, GTX118619, Genetex, Irvine, CA, USA) cells were counted along the whole retinal section, and the number was divided by the length of the retinal section to calculate the cell density. Numbers of neuronal nuclei (NeuN)^+^ cells in the ganglion cell layer (GCL) or calretinin^+^ cells in both the inner nuclear layer (INL) and GCL were also counted to estimate the survival of inner retinal neurons other than RGCs. To measure the fluorescent intensity of post-synaptic density-95 (PSD95), GFAP, or ionized calcium binding adaptor molecule 1 (Iba1) staining in the retinal sections, different groups of sections were processed using identical immunostaining and imaging parameters, and the mean fluorescent intensity in the retina was measured along the center, middle, and peripheral regions (approximately 500, 1000, and 1500 µm away from the optic disc, respectively) using ImageJ. For PSD95, the outer plexiform layer (OPL; region size 28 × 320 µm) and inner plexiform layer (IPL; region size 92 × 320 µm) were selected and their mean intensities were measured using Zen software (blue version, Carl Zeiss) and grouped into statistics based on different centrifugal degrees.

To assess the structure of the optic nerve, three areas along the optic nerve were photographed using confocal microscope, with a size of 320 × 320 µm. The three regions were located near the optic disc (proximal), in the middle of the optic nerve (middle), and far away from the optic disc (distal). The fluorescent intensity was measured using Zen software.

### Western blotting

To isolate total retinal proteins, the retinas were homogenized and lysed using a radio-immunoprecipitation assay buffer supplemented with a mix of phosphatase and protease inhibitors. The protein concentration was then determined using the BCA protein assay kit from Beyotime (P0011, Shanghai, China). Subsequently, equal quantities of protein (25 μL, 5 μg/μL) from each sample were resolved by electrophoresis on a 12% (v/v) SDS-PAGE gel and transferred onto a polyvinylidene difluoride membrane (Millipore, Bedford, MA, USA). The membrane was blocked with 5% bovine serum albumin in Tris-buffered saline with Tween 20 at room temperature for 120 minutes, followed by overnight incubation with primary antibodies at 4°C. The membrane was then incubated with secondary antibodies for 2 hours at room temperature. The antibody information is listed in **Table 2**. After six washes with 1 Tris-buffered saline with Tween 20 for 10 minutes each, the membrane was visualized using the enhanced chemiluminescence system (Bio-Rad, Hercules, CA, USA). Image analysis and semi-quantification were conducted using ImageJ software. The ratio of the total intensity of the tested protein to that of the reference protein was computed and then normalized to the mean value of the control group. Each experiment was repeated at least three times.

**Table 2:** Antibodies used for western blot assay

Antibody	Supplier	Cat#	RRID	Dilution
**Primary antibody**				
Chicken anti-GFAP	Aves Labs, Davis, California, USA	GFAP5727980	AB_2313547	1:1000
Rabbit anti-BDNF	Invitrogen	PA5-85730	AB_2792869	1:1000
Rabbit anti-GAPDH	CST, Danvers, Massachusetts, USA	5174S	AB_10622025	1:1000
Rabbit anti-GDNF	Abcam	ab176564	AB_2936981	1:1000
Rabbit anti-β-tubulin	CST	2146S	NA	1:2500
**Secondary antibody**				
Goat-anti-rabbit HRP-conjugated	Jackson ImmunoResearch	111-035-045	AB_2337938	1:2500
Goat anti-chicken HRP-conjugated	Abcam	ab6877	AB_955465	1:5000

BDNF: Brain-derived neurotrophic factor; GAPDH: glyceraldehyde 3-phosphate dehydrogenase; GDNF: glial cell-derived neurotrophic factor; GFAP: glial fibrillary acidic protein; HRP: horseradish peroxidase.

### Quantitative reverse transcription-polymerase chain reaction

Quantitative reverse transcription-polymerase chain reaction (qRT-PCR) was used to evaluate changes in the expression of genes related to nutritional factors and inflammation markers. Each retina was dissected and immersed in TRIzol™ Reagent (15596026, Invitrogen, Carlsbad, CA, USA) for RNA extraction according to the manufacturer’s instructions. RNA concentration and purity were determined using a NanoDrop2000c spectrophotometer (Thermo Fisher Scientific). cDNA was synthesized using a PrimeScript^TM^ RT reagent Kit with gDNA Eraser (Perfect Real Time) from Takara (Beijing, China). The primers for qRT-PCR were designed based on the primer bank and were synthesized by Sangon Biotech (Shanghai, China). A list of the primers used in the present study is provided in **Table 3**. qRT-PCR was performed using TB Green® Premix Ex Taq^TM^ II (Tli RNaseH Plus) (Takara) on a LightCycler480 System (Roche, Basel, Switzerland), following the manufacturer’s protocol. Glyceraldehyde 3-phosphate dehydrogenase served as the internal control, and fold changes were calculated using the comparative Ct method.

**Table 3:** Quantitative reverse transcription-polymerase chain reaction primers for quantification of gene expression

Target	Sequence (5' to 3')	Product size (bp)
*Gapdh*	Forward: CAT GGC CTT CCG TGT TCC TA	20
	Reverse: CCT GCT TCA CCA CCT TCT TGA T	22
*Gdnf*	Forward: TGC CAG AGG ATT ACC CAG AT	20
	Reverse: AGG TCA TCG TCA TAG GCT GT	20
*Gfap*	Forward: TTC AGC CAC ACC TTT CCA GC	20
	Reverse: CCT TAG AGG AGG CCT GGG AG	20
*Bdnf*	Forward: TCA TAC TTC GGT TGC ATG AAG G	22
	Reverse: AGA CCT CTC GAA CCT GCC C	19
*Il1b*	Forward: TTG AAG TTG ACG GAC CCC AA	20
	Reverse: TGT TGA TGT GCT GCT GCG A	19
*Nfκb*	Forward: ACA GAG GCC ATT GAA GTG A	19
	Reverse: CGT GGA GGA AGA CGA GAG	18
*Tnfa*	Forward: CAC AAA CCA CCA AGT GGA GGA	21
	Reverse: ACA AGG TAC AAC CCA TCG GCT	21
*Il6*	Forward: TTC CAT CCA GTT GCC TTC TTG	21
	Reverse: CAT TTC CAC GAT TTC CCA GAG	21

Bdnf: Brain-derived neurotrophic factor; Gapdh: glyceraldehyde 3-phosphate dehydrogenase; Gdnf: glial cell–derived neurotrophic factor; Gfap: glial fibrillary acidic protein.

### Statistical analysis

No statistical methods were used to predetermine sample sizes; however, our sample sizes were similar to those reported in a previous publication (Liu et al., 2021). All data are presented as the mean ± standard error of the mean. Statistical analyses were conducted using Prism 9 software (GraphPad Software, Boston, MA, USA). The Shapiro–Wilk test was used to assess normality. For parametric data, significance was determined using Student’s *t*-test or one- or two-way analysis of variance followed by Tukey’s *post hoc* test or Sidak’s multiple comparison test. For non-parametric data, the Mann–Whitney *U* test or Kruskal–Wallis test with Dunn’s *post hoc* analysis was used. *P* < 0.05 was considered significant. Unless specified otherwise, *n* represents the total number of mice examined per group, and one batch of experiments with multiple animals was performed.

## Results

### Specific neurogenic differentiation factor 1 overexpression in Müller cells

We first examined how quickly the AAV-GFAP-ND1-GFP virus was able to induce ND1 overexpression in the normal retina, to optimize treatment timing after ONC injury.

**Figure 1:**
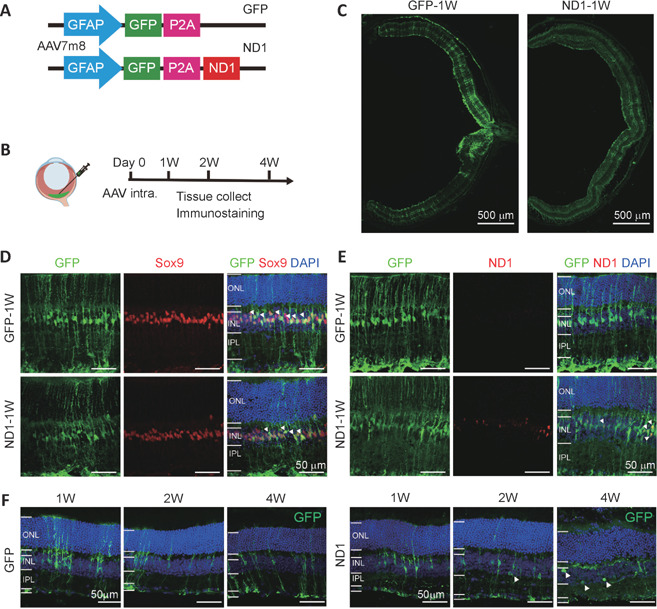
Specific overexpression of neurogenic differentiation factor 1 (ND1) in retinal Müller cells by adeno-associated virus (AAV) infection. (A) Construct of the AAV carrying green fluorescent protein (GFP; control) or ND1. (B) Experimental protocol. AAV was intravitreally injected into the eyes of adult C57 mice, and retinal tissue was collected for immunostaining at 1, 2, and 4 weeks (W) after injection. (C) Retinal sections showing a large area infected by the AAV; AAV can therefore effectively infect the retina. Green shows GFP expression. (D) Immunostaining of GFP and SOX-9 (a marker of Müller cells) on retinal sections. All GFP^+^ cells were co-stained with SOX-9 (examples indicated by arrowheads). (E) Immunostaining of GFP and ND1 on retinal sections. ND1 expression in GFP^+^ cells (examples indicated by arrowheads) were observed in retinas infected with ND1 virus but not GFP control virus. (F) Images of retinal sections showing the GFP^+^ cells over time after virus injection. In the ND1-infected retina, some GFP^+^ neuron-like cells appeared (white arrowheads), whereas other cells exhibited the morphology of amacrine cells (with somas in the deep inner nuclear layer [INL]), and others showed characteristics of horizontal cells (with wide branches in the outer plexiform layer [OPL] and soma in the top INL). GCL: Ganglion cell layer; IPL: inner plexiform layer.

The AAV-GFAP-ND1-GFP (or control virus, **Figure 1A**) was intravitreally injected into normal mice, and the specific expression of ND1 over time was examined using immunostaining (**Figure 1B**). By 1 week post-injection (1 wpi), a large area of the retina was infected in both the control (GFP) and ND1 groups (**Figure 1C**). For both groups, the GFP^+^ cells showed clear Müller cell morphology; they were characterized by long processes that extended across the retina and had a soma located centrally within the INL. All GFP^+^ somas also expressed the transcription factor SOX-9, a marker of Müller cells (**Figure 1D**), indicating the specific infection of Müller cells. Immunostaining for ND1 further confirmed the overexpression of ND1 in GFP^+^ cells, whereas no ND1 staining was observed in the control group (**Figure 1E**). Therefore, AAV-GFAP-ND1-GFP injection can specifically induce ND1 overexpression in Müller cells within 1 week after virus injection.

At 2 weeks, and particularly at 4 weeks, the infection of Müller cells by ND1 also induced some GFP^+^ neuron-like cells in the GCL and INL, including horizontal cells and amacrine cells (**Figure 1F**). Notably, when 4-week GFP^+^ cells were compared between the control and ND1 groups, the cell morphology was completely changed in the ND1 group, suggesting Müller cell conversion. Indeed, by double staining retinal sections with neuronal markers, some GFP^+^ cells were observed to co-express NeuN (neuron marker) and RBPMS (a marker of RGCs) at 1 week post-injection (**Additional Figure 1**), consistent with our previous report (Xu et al., 2023). By contrast, no GFP^+^ neurons were detected in the control group. For the remaining experiments, we therefore mainly focused on a time course within 4 weeks.

### Neurogenic differentiation factor 1 overexpression improves retinal light responses of optic nerve crush-injured mice

**Figure 2:**
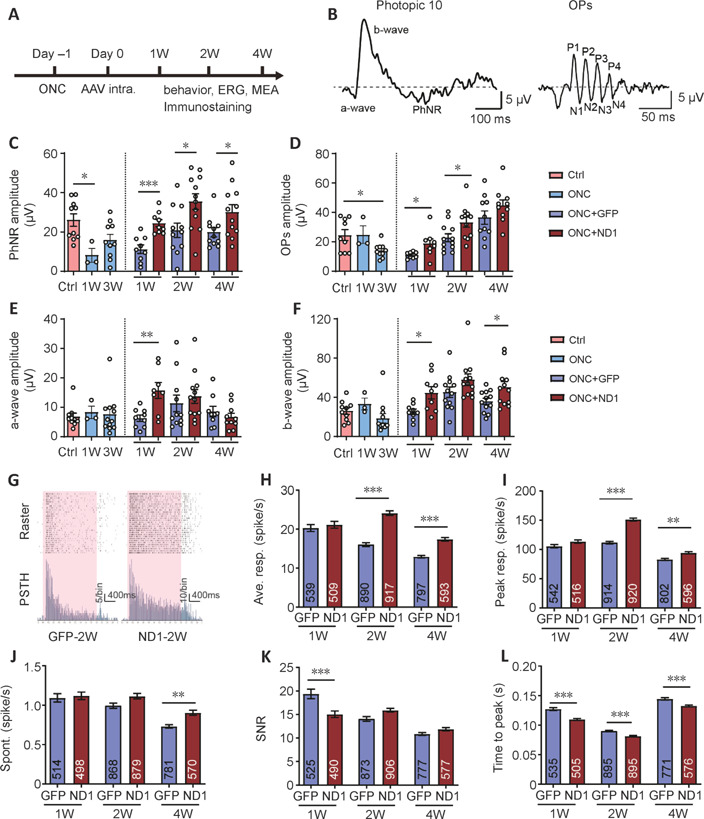
Neurogenic differentiation factor 1 (ND1) overexpression in Müller cells increases retinal light responses in optic nerve crush (ONC)–injured mice. (A) Experimental protocol. ONC injury was performed 1 day before the virus injection. At 1, 2, and 4 weeks post-injection (wpi), visual behavior testing, electroretinogram (ERG), and multi-electrode-array (MEA) recordings were performed. Some retinas were collected for immunostaining at 1 or 2 wpi. (B) Example traces of ERG responses from a normal mouse at photopic 10.0 cd·s/m^2^, with all peaks indicated. (C–F) The amplitudes of the photopic negative response (PhNR) (C), oscillatory potential (OP) peak (D), a-wave (E), and b-wave (F) at various times after ONC injury and after virus injection. ONC injury reduced the amplitudes of PhNR and oscillatory potentials in normal mice (left of the dotted line), and ND1 improved the inner retinal light responses compared with the green fluorescent protein (GFP) group (right of the dotted line). The dotted line separates two different batches of experiments, which underwent individual statistical comparisons. (G) Examples of the responses of two ON-type retinal ganglion cells (RGCs) to 2-second light stimuli (purple area) recorded using MEA in ONC-injured animals infected with GFP or ND1 virus. The top panel shows the raster plot, with each dot representing a spike. The bottom panel shows the peristimulus time histogram (PSTH), in a scale of the number of spikes/10 ms bin. Note the different scales of the GFP and ND1 groups. (H–J) The firing rate of average light responses (H), peak light responses (I), and spontaneous firing (J) in animals infected with GFP or ND1 at various times after infection. (K) The signal-to-noise ratios (SNRs) of RGCs firing in different groups. (L) Time to the peak of the PSTH for RGCs in different groups. ND1 overexpression increased the average and peak light responses compared with the GFP group. It also decreased the time to the peak of the PSTH. *P < 0.05, **P < 0.01, ***P < 0.001 (one-way analysis of variance followed by Tukey’s multiple comparison test). *n* = 10, 3, 12, 9, 8, 11, 12, 11, and 12 animals for the columns from left to right in C–F. Numbers in the bars indicate the numbers of RGCs recorded from at least three animals in each group for H–L. Ave.: Average; Ctrl: control; intra: intravitreal; resp.: response; Spont: spontaneous firing; W: week.

After identifying the timeline of ND1 overexpression, we applied a virus injection to the ONC injury model at 1 day post-surgery, which corresponds to the acute decline of RGC survival. We then examined visual behavior, retinal light responses, and retinal structure at 1, 2, and 4 wpi (**Figure 2A**).

Retinal light responses were first examined using electroretinogram recordings. A typical photopic response from a normal mouse is shown in **Figure 2B**. The amplitudes of the a-wave, b-wave, PhNR, and OPs were analyzed (**Figure 2B**). Compared with control mice, ONC significantly reduced the amplitudes of the PhNR and OPs, indicating that inner retinal neurons are impaired by central retinal artery injury during ONC (**Figure 2C** and **D**). The overexpression of ND1 significantly improved the amplitudes of the PhNR and OPs across time (**Figure 2C** and **D**). Furthermore, although ONC had little effect on the amplitudes of a- and b-waves, ND1 infection further improved the response amplitudes of a- and b-waves at 1 wpi (**Figure 2E** and **F**). Together, our findings suggest that ND1 infection enhances retinal light responses across time and cell types.

We next used MEA to evaluate the spiking of individual RGCs. An example of the spiking of one ON-RGC (whose spiking increased at light ON) from each group is shown as a raster plot and PSTH (**Figure 2G**) at 2 wpi. ND1 infection significantly increased the average responses (**Figure 2H**) and peak responses (**Figure 2I**) from 2 wpi compared with the GFP control group, and this effect remained at 4 wpi. ND1 also significantly increased spontaneous firing at 4 wpi (*P* < 0.01) (**Figure 2J**). The signal-to-noise ratio was decreased at 1 wpi after ND1 infection (*P* < 0.001 *vs*. GFP), and was then gradually restored at 2 and 4 wpi (**Figure 2K**). The time to peak was greatly decreased at all time points after ND1 infection (**Figure 2L**). These results indicate that ND1 overexpression increases the light responses of individual RGCs.

**Figure 3:**
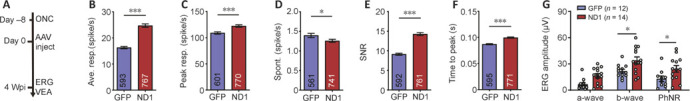
Neurogenic differentiation factor 1 (ND1) overexpression at the chronic stage of optic nerve crush (ONC) injury increases retinal light responses. (A) Experimental protocol. Retinas were subjected to virus injection at 8 days after ONC, and were recorded at 4 weeks post-injection (wpi). (B–F) Firing properties of individual retinal ganglion cells (RGCs) recorded using multi-electrode array (MEA). (G) Electroretinogram (ERG) amplitudes. *P < 0.05, ***P < 0.001 (Student’s *t*-test for B–F, one-way analysis of variance followed by Tukey’s multiple comparison test for G). Numbers in the bars indicate the numbers of RGCs recorded from at least three animals in each group for the MEA data. Ave.: Average; PhNR: photopic negative response; resp.: response; SNR: signal to noise ratio; Spont.: spontaneous firing.

We then examined whether ND1 can restore light responses if the virus is injected at day 8 after ONC, when over 60% of RGCs died and reached the chronic decay course (**Figure 3A**). The MEA recordings from injured animals revealed significant improvements in the average and peak responses of RGCs at 4 weeks after ND1 infection. ND1 further reduced the spontaneous firing of RGCs while increasing both signal-to-noise ratio and latency (**Figure 3B–F**). Significant improvements in b-wave and PhNR responses were also observed at 4 wpi with ND1 (**Figure 3G**).

Taken together, our findings indicate that ND1 overexpression in Müller cells enhances light responses in individual RGCs and the whole retina when infection occurs at both acute and chronic decay times.

### Neurogenic differentiation factor 1 slightly improves retinal structure in the optic nerve crush–injured mouse

**Figure 4:**
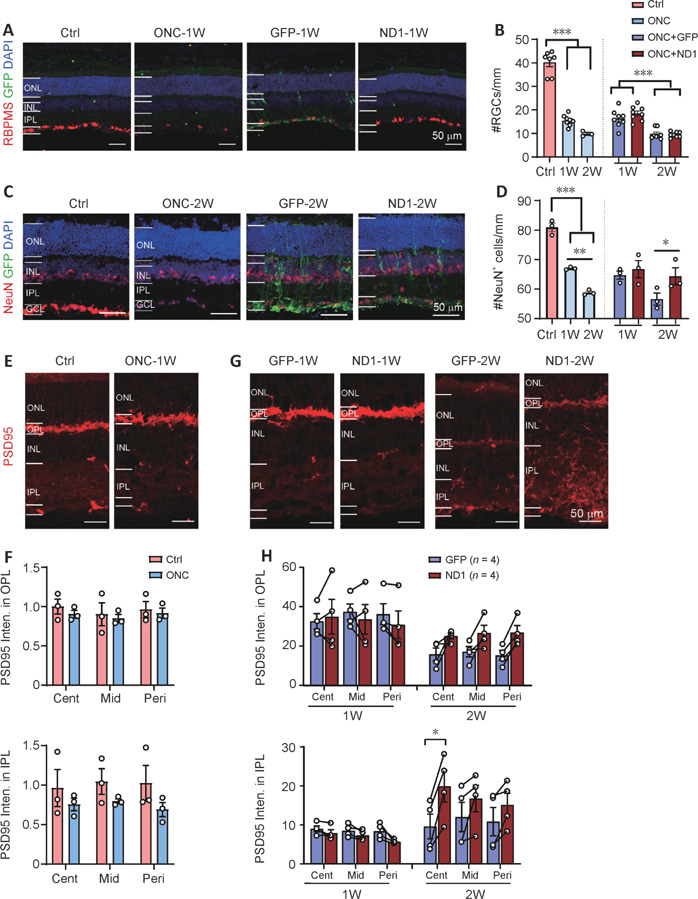
Neurogenic differentiation factor 1 (ND1) overexpression in Müller cells slightly improves inner retinal structure in optic nerve crush (ONC)–injured mice. (A, C) Images of retinal sections stained with the retinal ganglion cell (RGC) marker RNA-binding protein with multiple splicing (RBPMS) (A) and the neuronal marker NeuN (C) at 1 or 2 weeks post-injection (wpi), with normal and ONC-injured groups as controls. (B, D) The densities of RGCs (B) and neurons in the ganglion cell layer (GCL) (D) of different groups. ONC injury reduced the densities of RGCs (B, left of dotted line) and NeuN^+^ cells (D, left of dotted line). ND1 did not increase the density of RGCs (B, right of dotted line) but slightly increased NeuN^+^ cell density (D, right of dotted line). The dotted line separates two different batches of experiments, which underwent individual statistical comparisons. (E, G) Images of retinal sections stained with post-synaptic density 95 (PSD95), with normal control and ONC-injured groups in E and virus-injected groups in different batches of experiments in G. (F, H) The fluorescent intensity (Inten.) of PSD95 in the outer plexiform layer (OPL) and inner plexiform layer (IPL) of the retina in the center (Cent), middle (Mid), and peripheral (Peri) regions for different groups. Different batches of experiments were performed for F and H. ND1 increased PSD95 expression in the center region at 2 wpi. Data from eyes of the same animals (one infected with GFP and the other infected with ND1) are connected with lines. *n* = 7, 8, 3, 8, 8, 8, and 8 animals for the columns shown in B from left to right. *n* = 3 animals for each column in D and F. *n* = 4 animals for each column in H. *P < 0.05, **P < 0.01 (one-way analysis of variance followed by Tukey’s multiple comparison for B, D, F; two-way analysis of variance followed by Sidak’s multiple comparison for H). Ctrl: control; INL: inner nuclei layer; ONL: outer nuclei layer; w: week(s).

Because ND1 overexpression improved the retinal light responses, we next examined retinal structure by immunostaining for markers of RGCs, amacrine cells, and synapses. RGC survival was first examined by immunostaining for RBPMS; examples from 1 wpi are shown in **Figure 4A**. ONC injury greatly reduced RGC survival to only 38% of control levels at 1 wpi and 24% of control levels at 2 wpi (**Figure 4B**). However, ND1 overexpression in Müller cells did not improve RGC survival within 2 weeks (**Figure 4B**).

Given that ONC surgery impairs inner retinal neurons, including RGCs and amacrine cells, we next used NeuN staining to assess the survival of inner neurons (**Figure 4C**). Similar to RGCs, the survival of NeuN^+^ cells in the GCL was greatly reduced by ONC injury, to 83% of control levels at 1 wpi and 73% of control levels at 2 wpi (**Figure 4D**). Cell numbers increased slightly after ND1 injection and reached a significant difference at 2 wpi (*P* < 0.05, 70% of control in GFP *vs*. 79% of control in ND1, **Figure 4D**).

Next, we evaluated the inner retinal structure at 1 and 2 wpi by immunostaining for calretinin^+^ amacrine cells (**Additional Figure 2A**), whose survival decreased after ONC. At either 1 or 2 wpi, ND1 infection did not improve amacrine cell survival (**Additional Figure 2B**) or IPL thickness (**Additional Figure 2C**). These findings indicate that other types of amacrine cells, rather than calretinin^+^ cells, may account for the increased survival of neurons in the GCL.

We next explored the expression of synaptic connections in both the OPL and IPL (**Figure 4E**). ONC injury tended to reduce PSD95 fluorescent intensity in the IPL but not the OPL across all regions (**Figure 4E** and **F**), although there were no significant differences. Furthermore, ND1 overexpression tended to improve the expression of PSD95 in both the IPL and OPL at 2 wpi; this enhancement in expression reached significance in the center region after ND1 infection at 2 wpi (**Figure 4G** and **H**). These findings suggest that ND1 infection improves synaptic connections in the inner retina after ONC injury.

### Neurogenic differentiation factor 1 barely improves the injured optic nerve structure after optic nerve crush injury in 2 weeks

**Figure 5:**
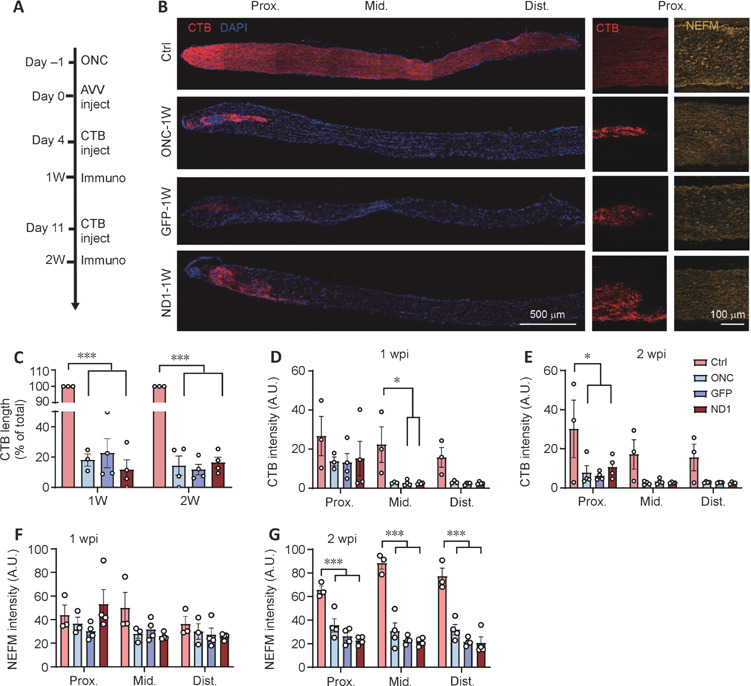
Neurogenic differentiation factor 1 (ND1) overexpression in Müller cells does not repair optic nerve structure. (A) Experimental protocol. Cholera toxin β (CTB) was intravitreally injected after optic nerve crush (ONC) and before sacrificing the animals. (B) Images of optic nerves with CTB labeling, with proximal regions enlarged to show CTB expression and neurofilament medium polypeptide (NEFM) staining. (C) The length of CTB signals as a percentage of full optic nerve length among different animal groups at 1 and 2 weeks post-injection (wpi). (D, E) The mean fluorescent intensities of CTB in the proximal (Prox.), middle (Mid.), and distal (Dist.) regions along the optic nerve at 1 (D) and 2 (E) wpi. (F, G) The mean fluorescent intensities of NEFM at 1 (F) and 2 (G) wpi. *n* = 3, 3, 4, 4, 3, 4, 4, and 4 animals for the columns from left to right in C. *n* = 3, 3, 4, and 4 for the four columns from left to right in each region (Prox., Mid., and Dist.) in D and F. *N* = 3, 4, 4, and 4 for the four columns from left to right in each region (Prox., Mid., and Dist.) in E and G. *P < 0.05, ***P < 0.001 (two-way analysis of variance followed by Tukey’s multiple comparison test). Ctrl: Control; immno: immunofluorescent staining; W: week(s).

After observing the ND1-induced improvements in retinal function and structure, we wondered whether ND1 overexpression in Müller cells may help to repair the damaged optic nerve after ONC injury. To track optic nerve projections, CTB was intravitreally injected 5 or 12 days after ONC (i.e., 3 days before sacrificing the animals at 1 or 2 wpi, respectively) (**Figure 5A**). CTB expression was measured at various regions along the optic nerve, and the structure of the remaining optic nerve fibers was further examined by immunostaining for neurofilament medium polypeptide (NEFM) (**Figure 5B**).

The intravitreal injection of CTB successfully labeled the whole optic nerve in control mice. By contrast, after ONC injury, CTB tracing was interrupted, and this interruption of CTB tracing was not improved by ND1 injection within 1–2 weeks (**Figure 5B**). The CTB length was greatly reduced by ONC injury; however, no differences were observed between the ONC and virus-injected groups (**Figure 5C**). The measurement of CTB intensity revealed greatly reduced CTB signals in the middle region of the optic nerve from 1 wpi, and in the proximal region of the optic nerve from 2 wpi after ONC. No differences were observed between the ONC and virus-injected groups (**Figure 5D** and **E**). For the remaining optic nerve fibers, no obvious changes were noted within 1 week after ONC (**Figure 5F**), and NEFM intensity was greatly reduced from 2 weeks after ONC compared with the normal control (**Figure 5G**). Overall, the impaired optic nerve structure was not improved by ND1 infection within a short time.

Consistent with the results of optic nerve structure, visual behaviors, which require visual signal transduction along the optic nerve to the cortex, were not improved by ND1 infection. Specifically, neither the black–white transition box nor the optomotor test showed any improvements in visual behaviors with ND1 (**Additional Figure 2D–G**). These results indicate that, although ND1 infection improves ONC-injured retinal function, it fails to rescue visual function transmitted by the optic nerve within a short time.

It is also worth noting that no GFP-labeled fibers were observed in the optic nerve, indicating that GFP^+^-labeled RGCs are unlikely to be able to project their axons into the optic nerve (at least within the 2 weeks that were examined in the present study).

### Neurogenic differentiation factor 1 overexpression does not reduce inflammation in the optic nerve crush-injured retina

We next explored the possible mechanism of ND1 overexpression in Müller cells. The overexpression of ND1 in astrocytes reportedly reduces inflammation in the injured brain (Zhang et al., 2020). We therefore first investigated the effects of ND1 on retinal inflammation.

**Figure 6:**
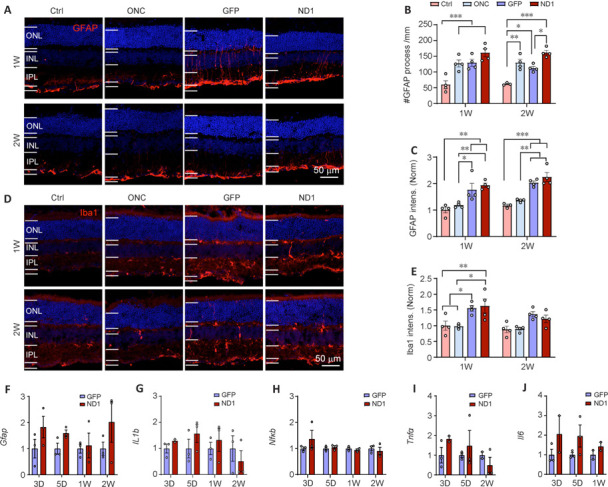
Neurogenic differentiation factor 1 (ND1) overexpression does not inhibit retinal inflammation in optic nerve crush (ONC)-injured retina. (A, D) Images of retinal sections stained with a marker of Müller processes, glial fibrillary acidic protein (GFAP) (A), or the microglial cell marker ionized calcium binding adaptor molecule 1 (Iba1) (D) at 1 and 2 weeks post-injection (wpi), with normal control and ONC-injured groups as controls. (B) Densities of GFAP^+^ processes per mm of the different groups at 1 and 2 wpi. (C) The mean intensities (Intens.) of GFAP fluorescence at 1 and 2 wpi. (E) The mean intensities of Iba1 fluorescence in retinas from different groups. (F) The expression of *Gfap* mRNA at 3 days post-injection (dpi), 5 dpi, 1 wpi, and 2 wpi. (G–J) The mRNA expression of the inflammatory cytokines *Il1b* (G), *Nfκb* (H), *Tnfα* (I), and *Il6* (J) in the ONC-injured retina at 3 dpi, 5 dpi, 1 wpi, and 2 wpi. No clear differences were noted between the ND1 and GFP groups. *N* = 4, 4, 4, 4, 3, 4, 4, and 4 for the columns from left to right in B and C. *n* = 4, 3, 4, 4, 4, 4, 4, and 4 for the columns from left to right in E. *n* = 3 for each column in F–J.*P < 0.05, **P < 0.01, ***P < 0.001 (two-way analysis of variance followed by Tukey’s multiple comparison test for B, C, and E, and Sidak’s multiple comparison test for F–J). Ctrl: Control; D: day; dpi: day post-infection; GCL: ganglion cell layer; INL: inner nuclei layer; IPL: inner plexiform layer; ONL: outer nuclei layer; OPL: outer plexiform layer; W: week(s).

Retinal inflammation is indicated by reactive gliosis in Müller cells and microglia. We thus examined the status of Müller cells and microglia by immunostaining for GFAP and Iba1, respectively (**Figure 6A** and **D**). Compared with the normal control, ONC injury induced a greater number of GFAP^+^ processes in Müller cells that crossed the whole retina (**Figure 6A**); however, GFAP intensity remained unchanged at both 1 and 2 wpi. The injection of each virus further increased GFAP expression in the ONC-injured groups. No changes were observed between the ND1 and GFP groups, except for more GFAP^+^ processes at 2 wpi (**Figure 6B** and **C**). GFAP expression was further examined by qRT-PCR (**Figure 6F**) and western blot analysis (**Additional Figure 3A** and **B**), and no changes were observed between the ND1 and control groups within 2 weeks.

Next, we quantified the expression of Iba1 using fluorescent intensity. In contrast to the increased GFAP expression, Iba1 expression did not increase after ONC compared with the control. The injection of either virus transiently increased Iba1 expression at 1 wpi but not 2 wpi. No differences were observed between the GFP and ND1 groups (**Figure 6D** and **E**).

Because virus infection induced ND1 overexpression within 1 week after infection, we next used qRT-PCR to explore the expression of a series of pro-inflammatory cytokines from 3 days post-injection (3 dpi). Within 2 weeks, ND1 overexpression had little effect on the mRNA expression levels of Il1b (**Figure 6G**), Nfkb (**Figure 6H**), Tnfα (**Figure 6I**), and Il6 (**Figure 6J**). Taken together, these findings indicate that ND1 overexpression does not inhibit inflammation in the ONC-injured retina at early stages after infection. Future studies should examine the longer-term effects of ND1.

### Neurogenic differentiation factor 1 overexpression transiently increases glial cell-derived neurotrophic factor levels

**Figure 7:**
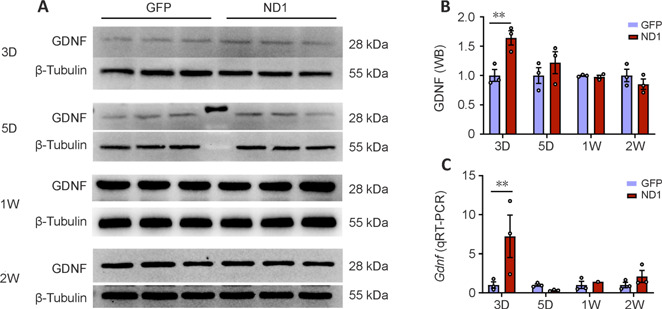
Neurogenic differentiation factor 1 (ND1) overexpression in Müller cells transiently increases glial cell-derived neurotrophic factor (GDNF) expression in optic nerve crush (ONC)-injured retina. (A) Western blot gels of GDNF protein from animals infected with green fluorescent protein (GFP) or ND1 at 3 days post-injection (dpi), 5 dpi, 1 week post-injection (wpi), and 2 wpi. Beta-tubulin served as the internal reference. (B, C) Normalized expression of GDNF protein (B) and mRNA (C) in retinas at various times post-infection. *n* = 3 for each column in B and C. **P < 0.01 (two-way analysis of variance followed by Sidak’s multiple comparison test). D: Day(s); qRT-PCR: quantitative reverse transcription-polymerase chain reaction; W: week(s); WB: western blotting.

Müller cells may exert a neuroprotective role by releasing nutrition factors, including glial cell-derived neurotrophic factor (GDNF) (Zhu et al., 2012) and brain-derived neurotrophic factor (BDNF) (Seki et al., 2003). We therefore explored the levels of GDNF in the retina using both western blot analysis and qRT-PCR. Western blot analysis revealed a weak but substantial increase in GDNF expression at 3 dpi, and the increased fold dropped over time (**Figure 7A** and **B**). As illustrated in **Figure 7C**, Gdnf mRNA increased significantly at 3 dpi before returning to the control level from 5 dpi onward. This finding is consistent with the observed increase in protein levels. Our data therefore indicate that ND1 transiently increases GDNF in the retina.

By contrast, no clear changes were observed in BDNF at all time points after ND1 infection (**Additional Figure 3C–E**). GDNF may therefore be the specific neurotrophic factor that is affected by ND1 overexpression in Müller cells.

## Discussion

In the present study, we identified the protective effects of Müller cell-specific ND1 overexpression on the ONC-injured retina. By specifically infecting Müller cells with AAV-GFAP virus carrying ND1 at an acute stage (1 day) after ONC injury, the light responses of RGCs and other retinal neurons were greatly enhanced as early as 1–2 wpi, and this effect lasted for up to 4 weeks. Infecting Müller cells at the chronic stage (8 days) after ONC also led to improved retinal function at 4 wpi. The rapid functional improvement may be caused by the increased release of GDNF from Müller cells, which slightly improved the survival of inner retinal neurons as well as synaptic connections in the IPL.

Müller cells produce many neuroprotective factors, such as basic fibroblast growth factor (Bringmann and Reichenbach, 2001), GDNF (Zhu et al., 2012), and BDNF (Seki et al., 2003), in response to trauma. These then activate downstream signaling pathways that promote cell proliferation, survival, or regeneration (Devoldere et al., 2019). In the current study, a transient increase in GDNF but not BDNF was observed at 3 days after Müller cells were infected with ND1. However, GDNF levels quickly dropped to those of controls by 5 dpi. This transient release of GDNF from Müller cells coincided with the timing of increased ND1 expression after virus injection, because our previous study identified ND1 expression in Müller cells at 5 dpi (Xu et al., 2023), and our qRT-PCR results also indicated the increased expression of ND1 as early as 3 dpi and peaking around 9 dpi (data not shown). However, it remains unclear why GDNF release is reduced at 5 dpi while ND1 expression continues to rise, and why GDNF but not BDNF has increased release. Furthermore, the transient enhancement of GDNF release cannot explain the long-term improvements in retinal light responses. Instead, the time-dependent reprogramming ability of Müller cells by ND1 infection, and the possible anti-inflammatory function of ND1 in the long term, may contribute to this effect.

The regenerative ability of Müller cells may also play a role in the functional recovery of the ONC-injured retina. In past decades, various strategies have been applied to reprogram Müller cells into retinal neurons in the injured or normal rodent retina, such as by activating the Wnt signaling pathway (Yao et al., 2016), inducing retinal injury by the intravitreal injection of N-methyl-D-aspartic acid (Karl et al., 2008) or via bright light exposure (Pavlou et al., 2024), overexpressing Ascl1 alone or with other transcription factors (Ueki et al., 2015; Todd et al., 2022), overexpressing Oxt2, Crx, and Nrl (Yao et al., 2018) or Math5 and Brn3b (Xiao et al., 2021), or knocking down Ptbp1 (Zhou et al., 2020). However, recent studies have begun to question the reprogramming abilities of Müller cells in mammals (Blackshaw and Sanes, 2021; Xie et al., 2022). Despite these concerns, our recent study indicated that in adult mouse retina, Müller cells can be reprogrammed into various types of retinal neurons using the same ND1-carrying virus as in the current study. Furthermore, the efficiency was limited in normal retinas but was enhanced under N-methyl-D-aspartic acid insult. In the present study, after ONC injury, a few GFP^+^ neuron-like cells indeed appeared as early as 1 wpi. Double staining of retinal sections revealed that these cells were positive for NeuN but negative for choline acetyltransferase (a marker of cholinergic amacrine cells) and RBPMS (a marker of RGCs). The numbers of GFP^+^ neuron-like cells was also limited within the first 2 weeks after virus infection. In the first 2 weeks, the regenerated neurons may therefore hardly aid in the fast functional improvement that occurs in the ONC-injured retina. Nonetheless, because the reprogramming of Müller cells by ND1 is time-dependent, with more Müller cells reprogrammed into neurons over a longer time than 2 weeks (Xu et al., 2023), we would still expect a contribution from Müller-regenerated neurons to long-term functional recovery.

Anti-inflammatory functions may also help retinal neurons to survive after injury. In multiple brain-injured models, ND1 overexpression in astrocytes is reported to inhibit inflammation (Zhang et al., 2020); we expected a similar effect of ND1 overexpression in Müller cells. However, we failed to observe any reduction in retinal inflammation within 2 wpi. By contrast, there was a tendency to further increase inflammation in the first 3 or 5 dpi. This seemingly contradictory effect may be caused by the different time points that were used after virus infection. ND1 may not immediately exert an anti-inflammatory effect following virus infection (as we observed in the current study), and it may take a relatively long time (for example, 4 wpi) for ND1 to trigger the anti-inflammatory function that has been observed in previous brain injury models.

Although ND1 overexpression improved retinal light responses in the present study, it did not repair the structure of the optic nerve. Consistent with this finding, the impaired visual behaviors were not improved by ND1, likely because an intact optic nerve is needed to transmit light information from the retina to the brain. The inability of ND1 to repair the optic nerve indicates that either the released GDNF was not transported to the injured site of the optic nerve, or the regenerative ability of Müller cells is too limited to be able to regenerate intact optic nerve fibers. Indeed, no virus GFP signal was observed in the optic nerve within 2 wpi, supporting the limited regeneration of optic nerve fibers. Alternatively, 2 weeks may be too short for any regenerated RGCs to extend their axons out of the eyeball and into the optic nerve. Nonetheless, given that there were no improvements in visual behaviors even by 4 weeks, which should be long enough for any regenerated axons to reach their brain targets, our findings indicate a limited regenerative ability of ND1.

There are a few limitations in the current study. First, we only examined retinal and optic nerve structure within 2 weeks after virus injection. It thus remains unclear whether more regenerated neurons from Müller cells may appear, and whether ND1 may exhibit anti-inflammatory effects, as it does in the brain, after 2 wpi. Searching for regenerated RGCs and examining inflammatory responses after 2 wpi may help to explain the persistent enhancements in retinal function after 2 wpi, because the enhanced GDNF release was only a transient effect. Second, we only examined retinal function within 4 weeks after virus injection. Whether ND1 can demonstrate long-term protective effects thus remains unclear. Third, although ND1 greatly improved the retinal light responses in the present study, retinal structure was only slightly improved. Improving ND1 expression in Müller cells using the enhanced promoter GFAP104 (Xu et al., 2023) may lead to improved retinal structure. Indeed, in the ND1-infected retina, only a few GFP+ cells expressed ND1, indicating the limited effects of ND1 overexpression with the current virus construct. In addition to using an enhanced promoter, ND1 expression may also be enhanced by changing the order of ND1 and GFP in the virus construct. On the other hand, infection by ND1 may affect the glial properties of Müller cells, which might then inhibit the GFAP promoter—and therefore the expression of both GFP and ND1 under the GFAP promoter—over time. The observed weaker GFP expression in the ND1 group than in the control group is consistent with this idea. Thus, simply modifying the viral construct may not significantly enhance ND1 expression because of the potential inhibitory effects of ND1 on the GFAP promoter.

In conclusion, the current study indicates that ND1 overexpression in Müller cells improves retinal function in the ONC-injured retina, and suggests that its neuroprotective effects may be caused by transiently enhanced GDNF release.

## Additional files:

***Additional Figure 1:*** *Neurogenic differentiation factor 1 (ND1) overexpression turns a limited number of Müller cells into retinal neurons.*

Additional Figure 1 Neurogenic differentiation factor 1 (ND1) overexpression turns a limited number of Müller cells into retinal neurons.
(A–C) Images of optic nerve crush (ONC)-injured retinal sections stained with green fluorescent protein (GFP) and NeuN (A), choline acetyltransferase (ChAT) (B), or RNA-binding protein with multiple splicing (RBPMS) (C), after being infected by ND1 for 1 week. Note that some GFP^+^ cells co-expressed the neuronal marker NeuN (yellow arrowheads) or the retinal ganglion cell (RGC) marker RBPMS (yellow arrowheads). There were also some GFP^+^ cells located in the ganglion cell layer (GCL) but they were not also positive for the amacrine cell marker ChAT or for RBPMS (white arrowheads).

***Additional Figure 2:*** *Neurogenic differentiation factor 1 (ND1) overexpression hardly improves amacrine cell survival and visual behaviors in optic nerve crush (ONC)-injured mice.*

Additional Figure 2 Neurogenic differentiation factor 1 (ND1) overexpression hardly improves amacrine cell survival and visual behaviors in optic nerve crush (ONC)-injured mice.
(A) Images of retinal sections stained with green fluorescent protein (GFP) and the amacrine cell marker calretinin, with normal and ONC-injured animals as the controls. (B) Densities of calretinin^+^ cells in the inner nuclear layer (INL) and ganglion cell layer (GCL) at 1 and 2 weeks post-injection (wpi), with normal and ONC-injured animals at 1 wpi as the controls. *n* = 6 and 2 animals for the control (Ctrl) and ONC groups, respectively, and 4 animals for the GFP and ND1 groups. (C) Thickness of the inner plexiform layer (IPL) at various distances away from the optic disk center in different animal groups. ND1 hardly affected amacrine cell density and IPL thickness. *n* = 4 for each column. (D, E) Illustration of the black–white transition box (D) and optomotor system (E). (F) Time spent by animals in the black box. *n* = 5 for all time points in the Ctrl and GFP groups, and 7, 6, and 7 for the 1 wpi, 2 wpi, and 4 wpi ND1 groups, respectively. (G) Visual acuities of animals at various times post-injury. ONC injury impaired visual behaviors, and ND1 did not improve them. *n* = 5 for all times points of Ctrl; *n* = 3, 5, and 4 for the 1, 2, and 4 wpi GFP groups, respectively; and *n* = 4, 6, and 6 for the 1, 2, and 4 wpi ND1 groups, respectively. *P < 0.05; **P < 0.01; two-way analysis of variance followed by Tukey’s multiple comparison test for B and Sidak’s multiple comparison test for C, F, and G.

***Additional Figure 3:*** *Neurogenic differentiation factor 1 (ND1) overexpression does not change glial fibrillary acidic protein (GFAP) or brain-derived neurotrophic factor (BDNF) expression over time.*

Additional Figure 3 Neurogenic differentiation factor 1 (ND1) overexpression does not change glial fibrillary acidic protein (GFAP) or brain-derived neurotrophic factor (BDNF) expression over time.
(A) Western blot gels of GFAP protein from animals infected with green fluorescent protein (GFP) or ND1 at 3 days post-injection (dpi), 5 dpi, 1 week post-injection (wpi), and 2 wpi. Glyceraldehyde 3-phosphate dehydrogenase (GADPH) served as the internal reference. (B) Normalized expression of GFAP protein in optic nerve crush (ONC)-injured retinas at various times post-infection. (C) West blot gels of BDNF protein from animals infected with GFP or ND1 at 3 dpi, 5 dpi, 1 wpi, and 2 wpi. Beta-tubulin served as the internal reference. (D, E) Normalized expression of BDNF protein (D) and mRNA (E) in retinas at various times post-infection. No differences were observed between the ND1 and GFP groups. *n* = 3 for each column.

## Data Availability

*All relevant data are within the paper and its Additional files.*
